# Supplementary material for: A Novel Multiprotein Bridging Factor 1-Like Protein Induces Cyst Wall Protein Gene Expression and Cyst Differentiation in Giardia lamblia
Source: Int J Mol Sci. 2021 Jan 29;22(3):1370. doi: 10.3390/ijms22031370 (PMC7866390; doi:10.3390/ijms22031370)
Supplement: Supplementary file 1 [file ijms-22-01370-s001.pdf]

Figure S1

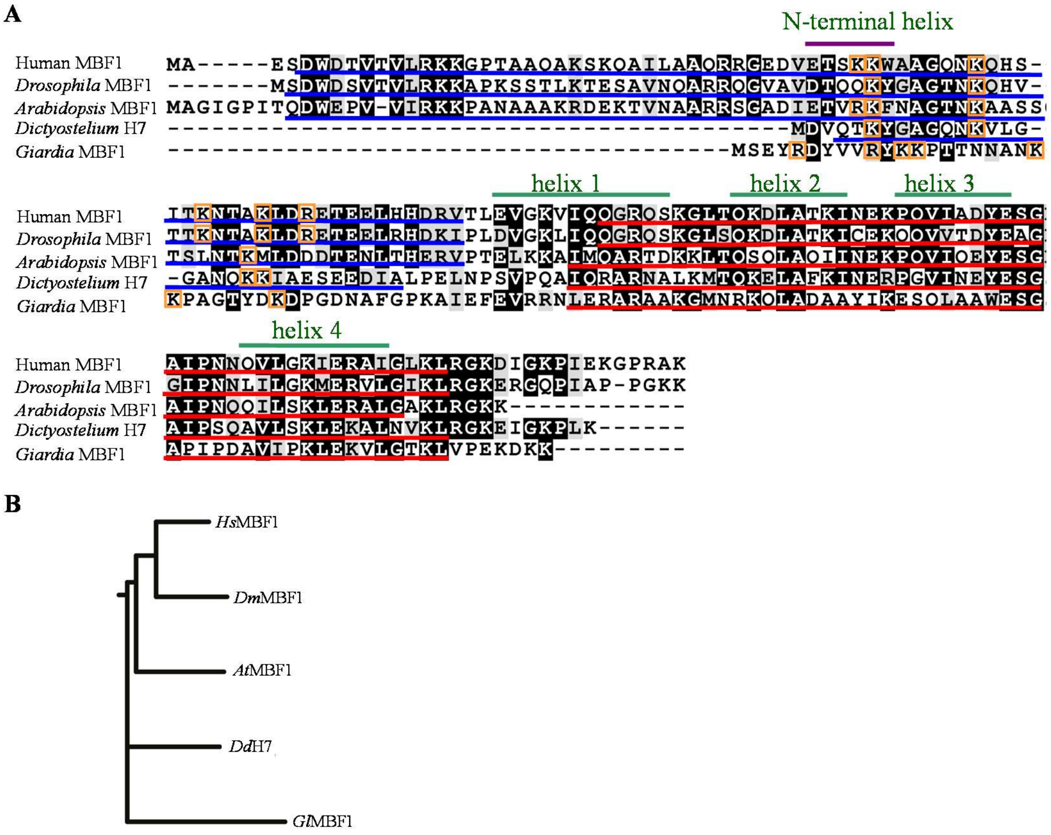
A
N-terminal helix
helix 1
helix 2
helix 3
helix 4
Human MBF1
Drosophila MBF1
Arabidopsis MBF1
Dictyostelium H7
Giardia MBF1
B
HsMBF1
DmMBF1
AtMBF1
DdH7
GlMBF1

Fig. S1. MBF1 sequence alignment and phylogenetic analysis. (A) Alignment of the MBF1 proteins. The MBF1 proteins from different organisms, including human, *Drosophila*, *Arabidopsis*, *Dictyostelium* and *Giardia*, are analyzed by ClustalW 1.83 with all default settings. GenBank *accession* numbers for human MBF1, *Drosophila* MBF1, *Arabidopsis* MBF1, *Dictyostelium* H7, and *Giardia* MBF1 are NP_003783.1, NP_524110.1, NP_565981.1, CAA33444.1, and XP_001704109.1, respectively. Black boxes, gray boxes and hyphens indicate identical amino acids, conserved amino acids and gaps in the respective proteins, respectively. The predicted HTH domains and MBF1 domains of various MBF1 proteins are indicated by red and blue underlines, respectively. No MBF1 domain was predicted for *Giardia* MBF1. The predicted N terminal α helix and basic amino acids of *Giardia* MBF1 are indicated by a purple line and orange boxes, respectively. (B) Phylogenetic analysis of the MBF1 proteins. A neighbor-joining phylogenetic tree was obtained from alignment of MBF1 proteins from various organisms as described. The bootstrap values determined from 1000 trees are not shown. Values are higher than 400.

Figure S2

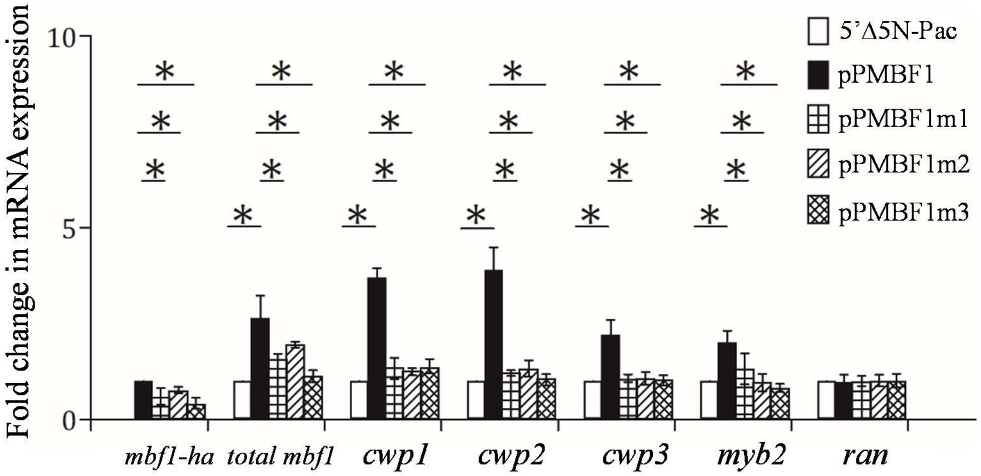

Fold change in mRNA expression
10
5
0
5'Δ5N-Pac
pPMBF1
pPMBF1m1
pPMBF1m2
pPMBF1m3
mbf1-ha
total mbf1
cwp1
cwp2
cwp3
myb2
ran

<u>Fig. S2.</u> Induction of *cwp1-3* and *myb2* gene expression in the MBF1-overexpressing cell line during encystation. The 5’Δ5N-Pac, pPMBF1, and pPMBF1m1-3 stable transfectants were cultured in encystation medium for 24h and then subjected to quantitative real-time RT-PCR analysis using primers specific for *mbf1-ha*, *mbf1*, *cwp1*, *cwp2*, *myb2*, *ran*, and 18S ribosomal RNA genes, respectively, as described in Fig. 3C.

Figure S3

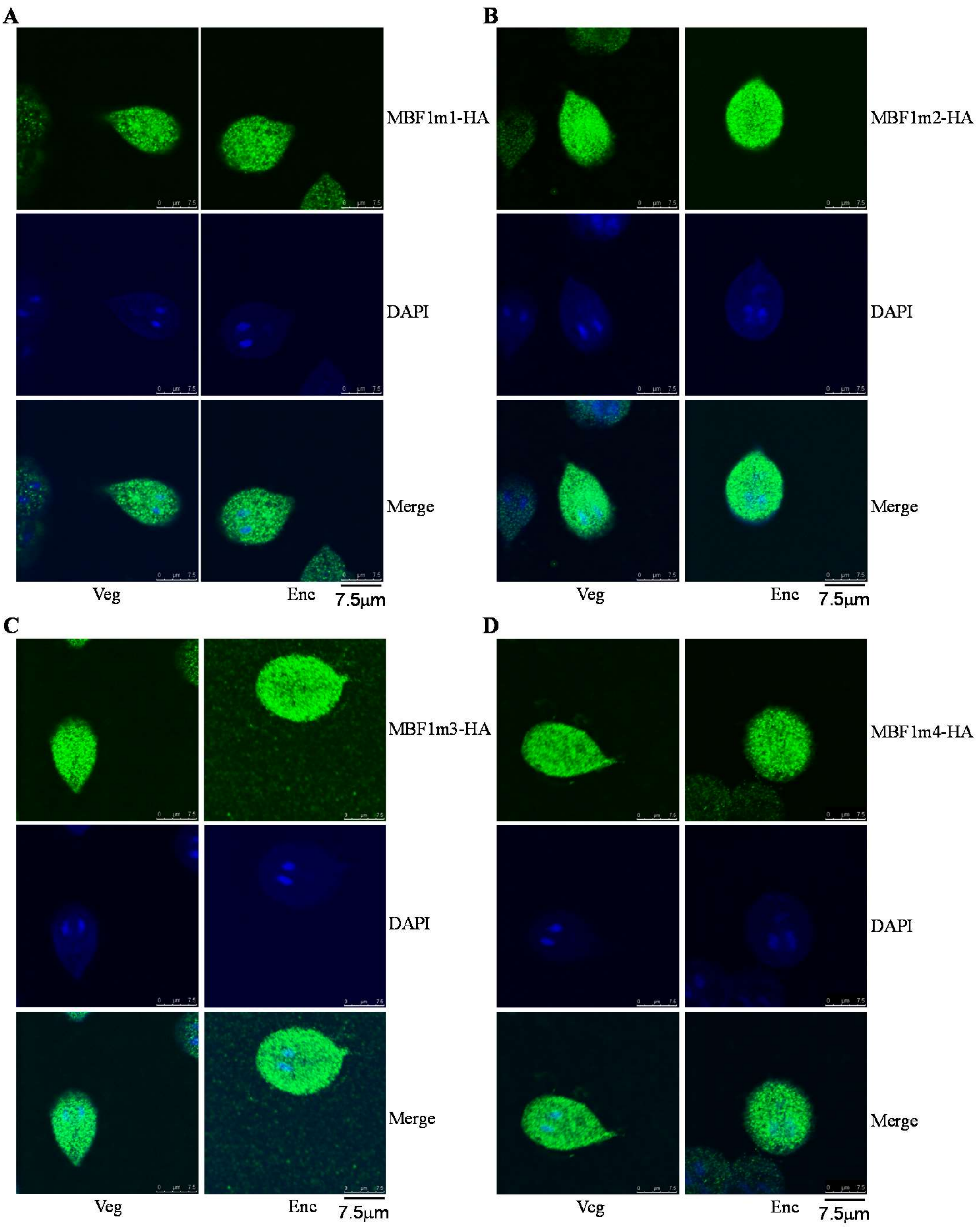
A
MBF1m1-HA
DAPI
Merge
Veg
Enc
7.5μm
B
MBF1m2-HA
DAPI
Merge
Veg
Enc
7.5μm
C
MBF1m3-HA
DAPI
Merge
Veg
Enc
7.5μm
D
MBF1m4-HA
DAPI
Merge
Veg
Enc
7.5μm

Fig. S3. **Immunofluorescence analysis of MBF1m1-4 distribution.** The pPMBF1m1-4 stable transfectants were cultured and then subjected to immunofluorescence analysis as described in Fig. 2E. The upper panels show the localization of the HA-tagged MBF1m1-4. The middle panels show the DAPI staining of cell nuclei. The bottom panels show the merged images.

Figure S4

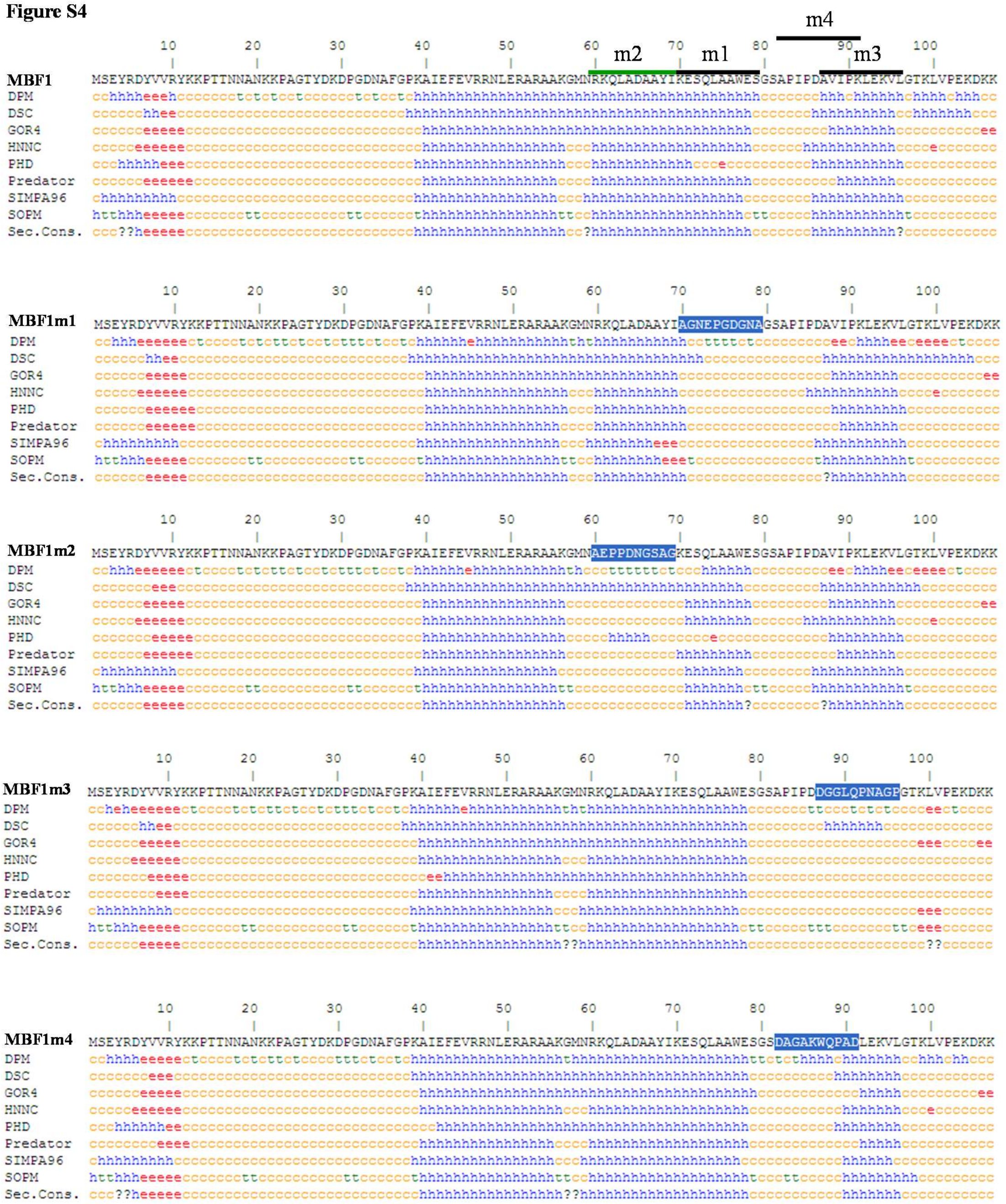

m4
m2
m1
m3
MBF1
MSEYRDYVVRYKKPTTNNANKKPAGTYDKDPGDNAFGPKAIEFEVRRNLERARAAKGMNRKQLADAAYIKESQLAAWESGSAPIPDAVIPKLEKVLGTKLVPEKDKK
MBF1m1
MSEYRDYVVRYKKPTTNNANKKPAGTYDKDPGDNAFGPKAIEFEVRRNLERARAAKGMNRKQLADAAYIAGNEPGDGNAGSAPIPDAVIPKLEKVLGTKLVPEKDKK
MBF1m2
MSEYRDYVVRYKKPTTNNANKKPAGTYDKDPGDNAFGPKAIEFEVRRNLERARAAKGMNAEPPDNGSAGKESQLAAWESGSAPIPDAVIPKLEKVLGTKLVPEKDKK
MBF1m3
MSEYRDYVVRYKKPTTNNANKKPAGTYDKDPGDNAFGPKAIEFEVRRNLERARAAKGMNRKQLADAAYIKESQLAAWESGSAPIPDDGGLQPNAGPGTKLVPEKDKK
MBF1m4
MSEYRDYVVRYKKPTTNNANKKPAGTYDKDPGDNAFGPKAIEFEVRRNLERARAAKGMNRKQLADAAYIKESQLAAWESGSDAGAKWQPADLEKVLGTKLVPEKDKK
DPM
DSC
GOR4
HNNC
PHD
Predator
SIMPA96
SOPM
Sec.Cons.

Fig. S4. Prediction of secondary structure of MBF1 mutants. The mutated region of MBF1m1-4 is indicated by blue boxes. The secondary structure of MBF1 and MBF1m1-4 was predicted by NPS analysis. H indicates the α-helix.

Figure S5

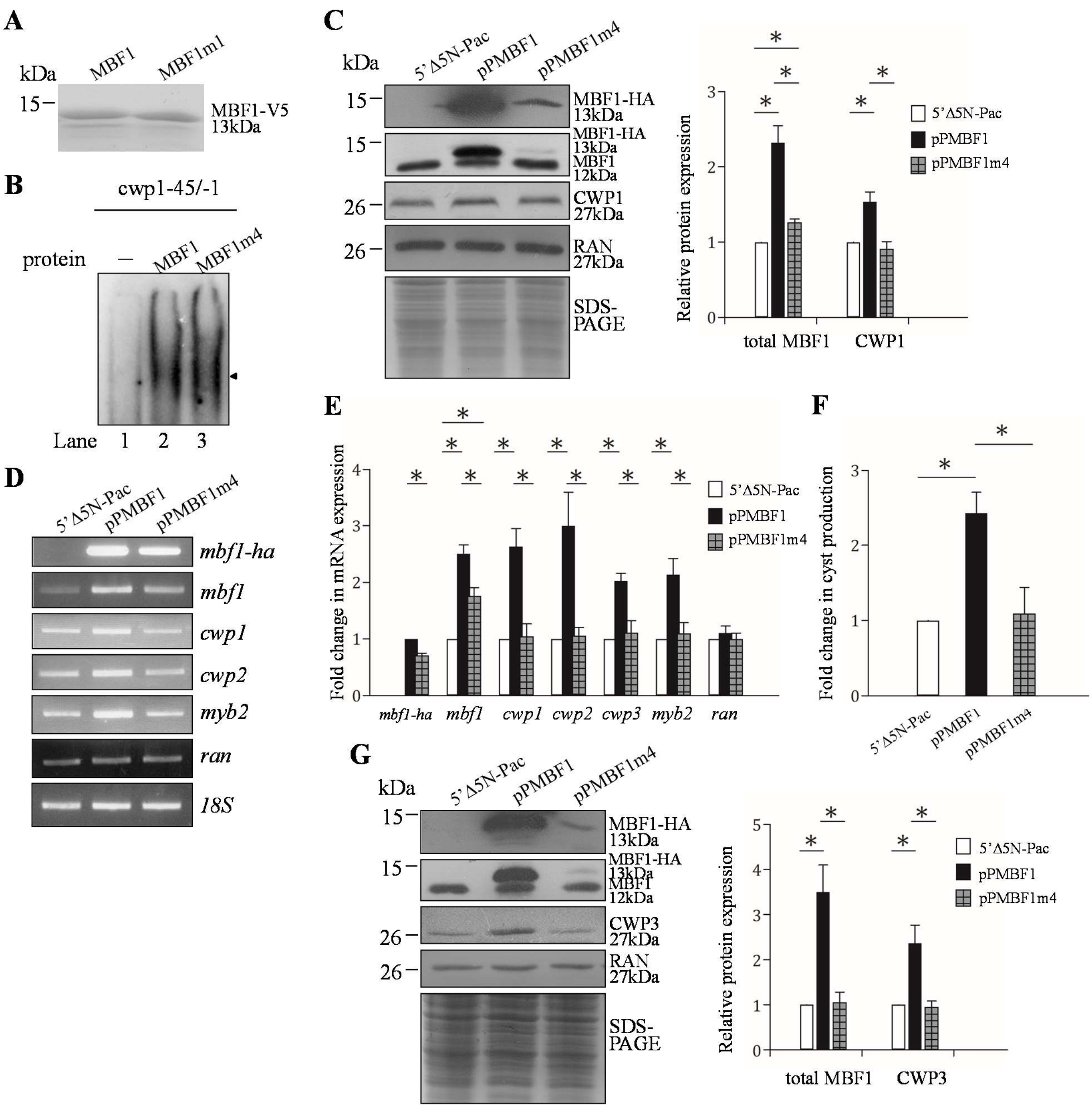
A
kDa
MBF1
MBF1m1
15
MBF1-V5
13kDa
B
cwp1-45/-1
protein
MBF1
MBF1m4
Lane 1 2 3
C
kDa
5'Δ5N-Pac
pPMBF1
pPMBF1m4
15
MBF1-HA
13kDa
MBF1
12kDa
26
CWP1
27kDa
RAN
27kDa
SDS-PAGE
Relative protein expression
total MBF1
CWP1
D
5'Δ5N-Pac
pPMBF1
pPMBF1m4
mbf1-ha
mbf1
cwp1
cwp2
myb2
ran
18S
E
Fold change in mRNA expression
mbf1-ha
mbf1
cwp1
cwp2
cwp3
myb2
ran
F
Fold change in cyst production
G
kDa
CWP3
27kDa
total MBF1
CWP3

Fig. S5. Analysis of MBF1m4. (A) SDS-PAGE analysis of recombinant MBF1 and MBF1m4 proteins. MBF1 or MBF1m4 protein with a C-terminal V5 tag was purified from *E. coli* by affinity chromatography and then analyzed by SDS-PAGE and silver staining. (B) MBF1m4 has DNA-binding ability. Electrophoretic mobility shift assays were performed using purified MBF1 and MBF1m4, and cwp1-45/-1 probe. The arrowhead indicates the shifted complex. . (C) Expression of MBF1m4 decreased the level of CWP1 protein. The 5'Δ5N-Pac, pPMBF1, and pPMBF1m4 stable transfectants were cultured in growth medium and then subjected to SDS-PAGE and Western blot. The blot was probed by anti-HA, anti-MBF1, anti-CWP1, and anti-Ran antibodies, respectively. The intensity of bands from three Western blot assays was quantified as described in Fig. 3A. (D) RT-PCR analysis of gene expression in the MBF1- and MBF1m4- expressing cell lines. The 5'Δ5N-Pac, pPMBF, and pPMBF1m4 stable transfectants were cultured in growth medium and then subjected to RT-PCR analysis using primers specific for *mbf1-ha*, *mbf1*, *cwp1*, *cwp2*, *myb2*, and 18S ribosomal RNA genes, respectively. (E) Quantitative real-time PCR analysis of gene expression in the MBF1- and MBF1m4- expressing cell lines. Real-time PCR was performed using primers specific for *mbf1-ha*, *mbf1*, *cwp1*, *cwp2*, *cwp3*, *myb2*, *ran*, and 18S ribosomal RNA genes, respectively, as described in Fig. 3C. (F) Expression of MBF1m4 decreased the level of cyst generation. The 5'Δ5N-Pac, pPMBF1, and pPMBF1m4 stable transfectants were cultured in growth medium and then subjected to cyst count as described under "Materials and Methods" and Fig. 3D. (G) Expression of MBF1m4 decreased the level of CWP3 protein during encystation. The 5'Δ5N-Pac, pPMBF1, and pPMBF1m4 stable transfectants were cultured in encystation medium for 24h and then subjected to SDS-PAGE and Western blot. The blot was probed by anti-HA, anti-MBF1, anti-CWP3, and anti-Ran

antibodies, respectively. The intensity of bands from three Western blot assays was quantified as described in Fig. 3A.

Figure S6

ATGGGCACCGAGTACAAGCCCACGGTGCGCCTCGCCACCCGCGACGACGTCCCCCGGGCCG
TACGCACCCTCGCCGCCGCGTTCGCCGACTACCCCGCCACGCGCCACACCGTCGACCCGGA
CCGCCACATCGAGCGGGTCACCGAGCTGCAAGAACTCTTCCTCACGCGCGTCGGGCTCGAC
ATCGGCAAGGTGTGGGTCGCGGACGACGGCGCCGCGGTGGCGGTCTGGACCACGCCGGAGA
GCGTCGAAGCGGGGGCGGTGTTCGCCGAGATCGGCCCGCGCATGGCCGAGTTGAGCGGTTC
CCGGCTGGCCGCGCAGCAACAGATGGAAGGCCTCCTGGCGCCGCACCGGCCCAAGGAGCCC
GCGTGGTTCCTGGCCACCGTCGGCGTCTCGCCCGACCACCAGGGCAAGGGTCTGGGCAGCG
CCGTCGTGCTCCCCGGAGTGGAGGCGGCCGAGCGCGCCGGGGTGCCCGCCTTCCTGGAGAC
CTCCGCGCCCCGCAACCTCCCCTTCTACGAGCGGCTCGGCTTCACCGTCACCGCCGACGTC
GAGTGCCCG**AAGGACCGCGCGACCTGG**TGCATGACCCGCAAGCCCGGTGCCCTCGAGTGAg
ggcttgtgcattgctactactgagtctacttgagggtggatgcatctacactctgacttcg
gctttgatctttcagacacctcaaaggctgtaactgtgtaaacagatgcgctttcttgcag
agataaccccttacaaagtctgcctatgaatgccttgtccttgctcacatgtaacagaacg
gcttagtcaagaggccattgaataattaaaccatgagaggcttcacgttcagcgtcggcgg
gggctgccggaatgctcctgtcgactttggaccgtgtagagcagggacttccacggtgata
gaggttctctttagctccacgctctctgtaagcgttaacgttagcatctcaaatttgccac
ggcacattgatgctgttcccaagcaatttattctcaatccctcgagcaagatgaatgtgaa
gttcacatgggtctcaaaggatactaaatttcaaatcctcaacgaaactctgaatgttgaa
agccagaagtcagtgcagcaccttgtgctcttgggcttagcaactgagaaggaactgaaca
tctttttttcggcatccaatgagaagctctactttcgtattttgggtcg**gcccgagacttt**
ctttgtcca

Fig. S6. Replacement of the *mbf1* gene with the *pac* gene in the MBF1td cell line confirmed by PCR and sequencing. Genomic DNA was isolated from MBF1td and control cell lines cultured in growth medium. PCR was performed using primers specific for *pac* (PCR2 in Fig. 8A), which are PCR2F for bold region 1 and PCR2R for bold region 2, to verify the integration of the *pac* gene into the correct region in genomic DNA. The sequence results obtained from the PCR2 product are shown as underlined letters. Capital letters indicate the coding sequence for *pac* gene, which starts at ATG and stops at TGA. This indicates the replacement of the *mbf1* gene with the *pac* gene. The region used to clone the *mbf1* 3' region into the pMBF1td plasmid for HR is shown in red, which is also between the sequence of MBF13XF and MBF13KR. The underlined and lower case letters, which are downstream and outside of the red region of MBF13XF and MBF13KR, indicate that HR occurred in the sequence of *mbf1* 3' region and that the *pac* gene was integrated in the genomic DNA.

Figure S7

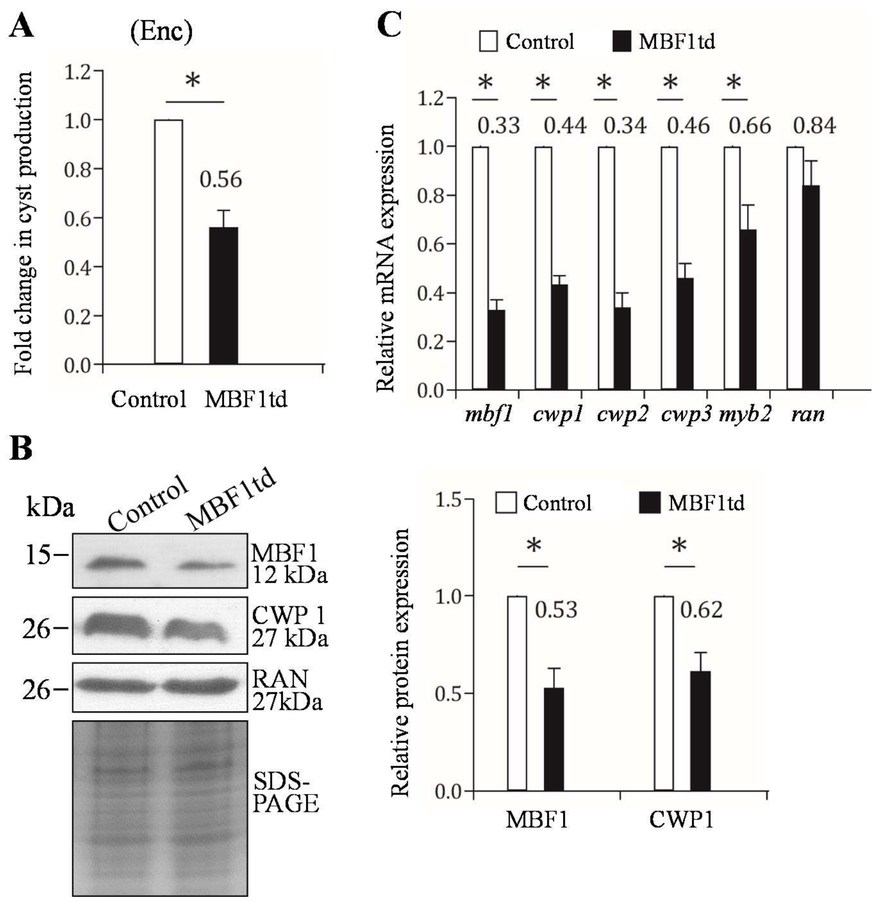
A
(Enc)
Fold change in cyst production
1.2
1.0
0.8
0.6
0.4
0.2
0.0
*
0.56
Control
MBF1td
B
kDa
Control
MBF1td
15–
MBF1
12 kDa
26–
CWP 1
27 kDa
26–
RAN
27kDa
SDS-
PAGE
C
Control
MBF1td
Relative mRNA expression
* * * * *
0.33 0.44 0.34 0.46 0.66 0.84
mbf1 cwp1 cwp2 cwp3 myb2 ran
Relative protein expression
1.5
1.0
0.5
0.0
* *
0.53
0.62
MBF1
CWP1

Fig. S7. Decrease in expression of *cwp1-3*, and *myb2* by targeted disruption of the *mbf1* gene during encystation. (A) Cyst generation decreased by targeted disruption of the *mbf1* gene in the MBF1td cell line during encystation. The control and MBF1td cell lines were cultured in encystation medium for 24h (Enc) and then subjected to cyst count as described under "Materials and Methods" and Fig. 3D. (B) Targeted disruption of the *mbf1* gene decreased the CWP1 level in the MBF1td cell line during encystation. The control and MBF1td cell lines were cultured in encystation medium for 24h and then subjected to SDS-PAGE and Western blot analysis as described in Fig. 3A. The blot was probed with anti-MBF1, anti-CWP1, and anti-RAN antibodies, respectively. The intensity of bands from three Western blot assays was quantified as described in Fig. 3A. (C) Decrease in expression of *cwp1-3*, and *myb2* by targeted disruption of the *mbf1* gene in the MBF1td cell line during encystation. The control and MBF1td cell lines were cultured in encystation medium for 24h and then subjected to quantitative real-time RT-PCR analysis using primers specific for *mbf1*, *cwp1*, *cwp2*, *cwp3*, *myb2*, *ran*, and 18S ribosomal RNA genes, respectively, as described in Fig. 2A.

Figure S8

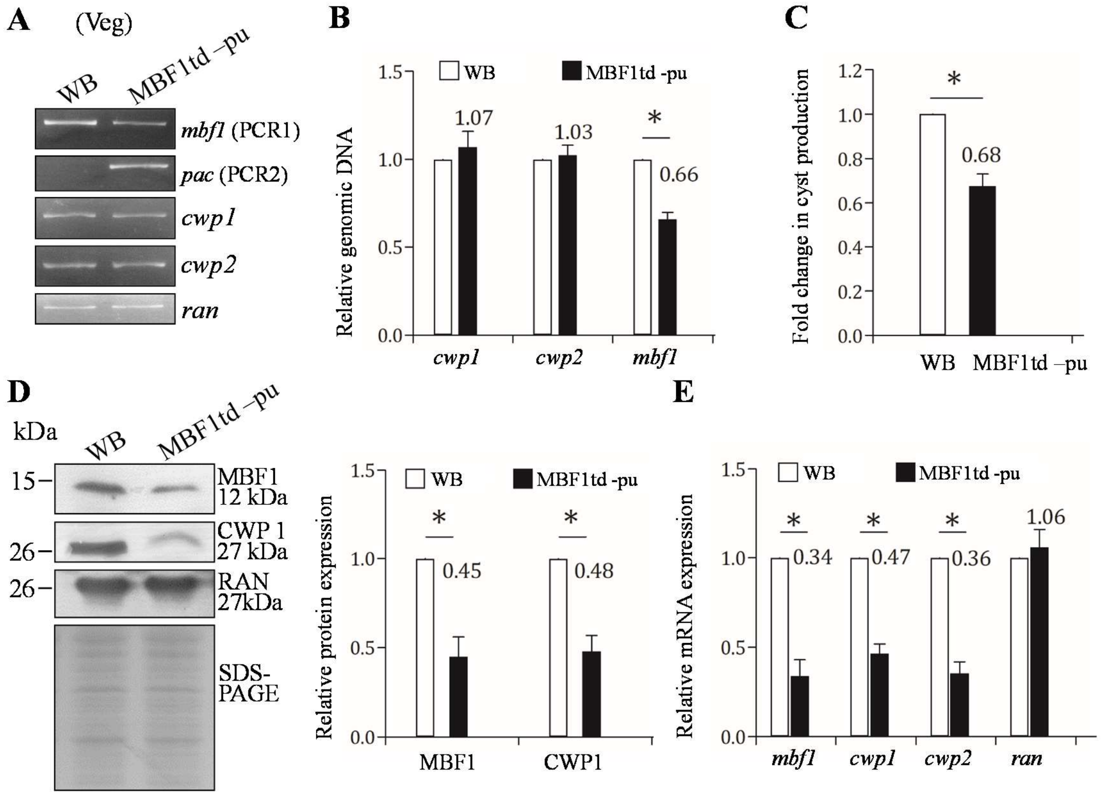

A
(Veg)
WB
MBF1td -pu
mbf1 (PCR1)
pac (PCR2)
cwp1
cwp2
ran
B
WB
MBF1td -pu
Relative genomic DNA
1.07
1.03
0.66
cwp1
cwp2
mbf1
C
Fold change in cyst production
0.68
WB
MBF1td –pu
D
kDa
15
26
26
MBF1
12 kDa
CWP 1
27 kDa
RAN
27kDa
SDS-PAGE
Relative protein expression
0.45
0.48
MBF1
CWP1
E
Relative mRNA expression
0.34
0.47
0.36
1.06
mbf1
cwp1
cwp2
ran

Fig. S8. Decrease in expression of *cwp1* and *cwp2* by targeted disruption of the *mbf1* gene after the removal of puromycin during vegetative growth. (A) Partial replacement of the *mbf1* gene with the *pac* gene in the MBF1 –pu cell line confirmed by PCR. Puromycin was removed from the MBF1td and control cell lines to obtain the MBF1td –pu and control –pu cell lines, respectively. Genomic DNA was isolated from MBF1td –pu and control -pu cell lines cultured in growth medium (vegetative growth, Veg). PCR was performed using primers specific for *mbf1* (PCR1), *pac* (PCR2), *cwp1*, *cwp2*, and *ran* genes, respectively, as described in Fig. 8B. (B) Partial disruption of the *mbf1* gene in the MBF1td -pu cell line confirmed by real-time PCR. Real-time PCR was performed using genomic DNA and primers specific for *mbf1*, *cwp1*, *cwp2*, and *ran* genes, respectively, as described in Fig. 8C. (C) Cyst generation decreased by targeted disruption of the *mbf1* gene in the MBF1td –pu cell line during vegetative growth. The control –pu and MBF1td –pu cell lines were cultured in growth medium and then subjected to cyst count as described under "Materials and Methods" and Fig. 3D. (D) Targeted disruption of the *mbf1* gene decreased the CWP1 level in the MBF1td –pu cell line during vegetative growth. The control –pu and MBF1td –pu cell lines were cultured in growth medium and then subjected to SDS-PAGE and Western blot analysis as described in Fig. 3A. The blot was probed with anti-MBF1, anti-CWP1, and anti-RAN antibodies, respectively. The intensity of bands from three Western blot assays was quantified as described in Fig. 3A. (E) Decrease in expression of *cwp1* and *cwp2* by targeted disruption of the *mbf1* gene in the MBF1td –pu cell line during vegetative growth. The control –pu and MBF1td –pu cell lines were cultured in growth medium and then subjected to quantitative real-time RT-PCR analysis using primers specific for *mbf1*, *cwp1*, *cwp2*, *ran*, and 18S ribosomal RNA genes, respectively, as described in Fig. 2A.

Figure S9

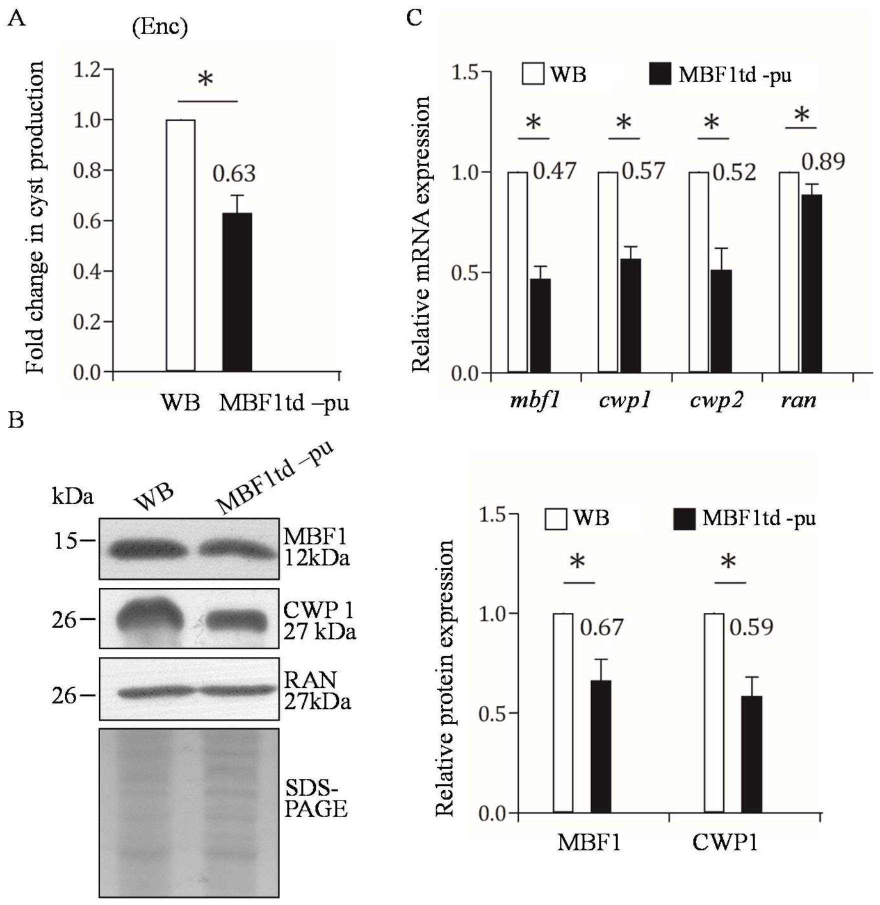

A
(Enc)
Fold change in cyst production
1.2
1.0
0.8
0.6
0.4
0.2
0.0
*
0.63
WB
MBF1td –pu
B
kDa
WB
MBF1td –pu
15
MBF1
12kDa
26
CWP 1
27 kDa
26
RAN
27kDa
SDS-
PAGE
C
Relative mRNA expression
1.5
1.0
0.5
0.0
WB
MBF1td -pu
*
0.47
0.57
0.52
0.89
mbf1
cwp1
cwp2
ran
Relative protein expression
0.67
0.59
MBF1
CWP1

__Fig. S9.__ **Decrease in expression of *cwp1* and *cwp2* by targeted disruption of the *mbf1* gene after the removal of puromycin during encystation.** (A) Cyst generation decreased by targeted disruption of the *mbf1* gene in the MBF1td –pu cell line during encystation. The control –pu and MBF1td –pu cell lines were cultured in encystation medium for 24h (Enc) and then subjected to cyst count as described under "Materials and Methods", as described in Fig. 3D. (B) Targeted disruption of the *mbf1* gene decreased the CWP1 level in the MBF1td –pu cell line during encystation. The control –pu and MBF1td –pu cell lines were cultured in encystation medium for 24h and then subjected to SDS-PAGE and Western blot analysis as described in Fig. 3A. The blot was probed with anti-MBF1, anti-CWP1, and anti-RAN antibodies, respectively. The intensity of bands from three Western blot assays was quantified as described in Fig. 3A. (C) Decrease in expression of *cwp1* and *cwp2* by targeted disruption of the *mbf1* gene in the MBF1td –pu cell line during encystation. The control –pu and MBF1td –pu cell lines were cultured in encystation medium for 24h and then subjected to quantitative real-time RT-PCR analysis using primers specific for *mbf1*, *cwp1*, *cwp2*, *ran*, and 18S ribosomal RNA genes, respectively, as described in Fig. 2A.

Table S1. Genes up or down regulated by MBF1 overexpression.

Number	Annotation	Orf number	Fold change (pPMBF1/5'Δ5N-Pac)*
1	VSP	41476	26.55
2	VSP	13402	21.92
3	VSP	14307	17.34
4	E2F1	23756	14.85
5	VSP	26590	11.23
6	VSP	13727	8.86
7	VSP	137614	8.16
8	VSP	105983	6.70
9	VSP	111873	6.02
10	VSP	114121	5.94
11	VSP, putative	96055	5.47
12	Hypothetical protein	117989	4.58
13	Hypothetical protein	105786	3.68
14	High cysteine membrane protein Group 1	25816	3.66
15	Hypothetical protein	9605	3.54
16	VSP, putative	114286	3.15
17	VSP	124980	3.02
18	VSP	15206	2.97
19	VSP, putative	92835	2.93
20	VSP	112113	2.48
21	Hypothetical protein	15125	2.29
22	Hypothetical protein	16078	2.23
23	VSP	114065	2.14
24	Hypothetical protein	8960	2.17
25	Dynein heavy chain	16804	2.10
26	Heat shock protein HSP 90-alpha	98054	2.06
27	Hypothetical protein	8505	1.95
28	Hypothetical protein	9210	2.02
29	Kinase, NEK	137733	2.02

30	Ubiquitin-activating enzyme E1 1	6288	2.01
31	DNA topoisomerase IIIα	7615	0.03
32	VSP	40591	0.17
33	VSP AS8	13194	0.17
34	VSP	114672	0.17
35	Hypothetical protein	99726	0.21
36	VSP with INR	119707	0.25
37	VSP	40571	0.26
38	VSP	90215	0.27
39	VSP	34357	0.27
40	VSP	105759	0.34
41	Hypothetical protein	123980	0.35
42	VSP with INR	40592	0.35
43	VSP	34196	0.38
44	VSP	115047	0.43
45	Hypothetical protein	114674	0.45
46	VSP	114653	0.46
47	VSP	116477	0.46
48	Hypothetical protein	26442	0.47
49	VSP	36493	0.47
50	Hypothetical protein	16697	0.48
51	Hypothetical protein	13239	0.49
52	VSP	112678	0.49
53	Elongation factor 1-gamma	12102	0.50

*p values were determined to be <0.05 for groups in which the average means changed by a factor of ≥2.0 or ≤0.5.

Table S2. Oligonucleotides used for construction of plasmids and PCR.

Name	Sequence (5'--->3')
mbf1F (PCR1F)	CACCATGTCCGAGTATCGTGACTAC
mbf1R (PCR1R)	CTTCTTATCCTTCTCTGGGAC
HAR	AGCGTAATCTGGAACATCGTATGGGTA
cwp1F	ATGATGCTCGCTCTCCTT
cwp1R	TCAAGGCGGGGTGAGGCA
cwp2F	ATGATCGCAGCCCTTGTTCTA
cwp2R	CCTTCTGCGGACAATAGGCTT
cwp3F	ATGTTTTCTCTGCTTCTTCT
cwp3R	TCTGTAGTAGGGCGGCTGTA
myb2F	ATGTTACCGGTACCTTCTCAGC
myb2R	GGGTAGCTTCTCACGGGGAAG
ranF	ATGTCTGACCCAATCAGC
ranR	TCAATCATCGTCGGGAAG
mbf1realF	CGACAAAGATCCCGGAGACA
mbf1realR	TTGCGACGCACTTCAAACTC
cwp1realF	AACGCTCTCACAGGCTCCAT
cwp1realR	AGGTGGAGCTCCTTGAGAAATTG
cwp2realF	TAGGCTGCTTCCCACTTTTGAG
cwp2realR	CGGGCCCGCAAGGT
cwp3realF	GCAAATTGGATGCCAAACAA
cwp3realR	GACTCCGATCCAGTCGCAGTA
myb2realF	TCCCTAATGACGCCAAACG
myb2realR	AGCACGCAGAGGCCAAGT
ranrealF	TCGTCCTCGTCGGAAACAA
ranrealR	AACTGTCTGGGTGCGGATCT
18SrealF	AAGACCGCCTCTGTCAATCAA
18SrealR	GTTTACGGCCGGGAATACG
mbf1KF	ACCGTGGTACCCCTCTCTTGACAAAGGAG

mbf1MR	CCGGCACGCGTCTTCTTATCCTTCTCTGG
top3βm1F	ATGCAGCGTATATCGCGGGGAATGAGCCGGGCGATGGAAATGCGGGCTCTGCTCCCAT
top3βm1R	ATGGGAGCAGAGCCCGCATTTCCATCGCCCGGCTCATTCCCCGCGATATACGCTGCAT
top3βm2F	CGAAGGGCATGAACGCGGAGCCGCCGGATAATGGGAGTGCGGGGAAGGAATCGCAGCT
top3βm2R	AGCTGCGATTCCTTCCCCGCACTCCCATTATCCGGCGGCTCCGCGTTCATGCCCTTCG
top3βm3F	CTCCCATTCCTGATGATGGTGGTCTTCAACCTAATGCTGGTCCTGGTACAAAGC
top3βm3R	GCTTTGTACCAGGACCAGCATTAGGTTGAAGACCACCATCATCAGGAATGGGAG
top3βm4F	GGGAATCCGGCTCTGATGCTGGTGCTAAATGGCAACCTGCTGATTTGGAGAAGGTCCT
top3βm4R	AGGACCTTCTCCAAATCAGCAGGTTGCCATTTAGCACCAGCATCAGAGCCGGATTCCC
mbf15HF	TCTGAAAGCTTCTCTAAAGT
mbf15NR	GGCGGCCATGGTTGTCACCAGGTCTTGAAAAC
mbf13XF	GGCGGCTCGAGTGATGAGGGCTTGTGCATTGCTACTAC
mbf13KR	GGCGGGGTACCGGATTGAGAATAAATTGCTTG
mbf1-guide	GAGAGCGGGTACCCTAGCTTATTGAAAAAGCGAGAGGCCATTGAGATGACTCGCCTGATTGCAATAGCAAACAGTGTCTATAGTCTAATTGTGGACAACAGAGGGCTTATTGCAACGTTGATGACCAAGTTCAACAAGGGCGTCGCCTCCTATGAGCAGGTCATTCGGAATTTCGACGACCGGTAGCGTCCCCAGAGTAAACCATTTTAAATTGAAATAGGCGGTTGGAAATAAAAGCGCGCC**GTTTGAAGTGCGTCGCA*ACC***GTTTTAGAGCTAGAAATAGCAAGTTAAAATAAGGCTAGTCCGTTATCAACTTGAAAAAGTGGCACCGAGTCGGTGCTTTTTTGAATTCGAGAGCG, underlined region is U6 promoter, bold region is for annealing, underlined and bold region is upstream 3nt of PAM, other region is scaffold RNA
PCR2F	AAGGACCGCGCGACCTGG
PCR2R	tggacaaagaaagtctcgggc
mbf15F	GTGAACTAAGAGGACTAGCCAG
mbf15R	CAGGTCTTGAAAACAAAGGTCTTC
18S5F	CCAAAAAAGTGTGGTGCAGG
18S5R	GCCGGGCGCGGGCGCCGCGG

cwp15F	CAACGGCTTACTAAATCATTCTCTTG
cwp15R	TTCTGTGTTTCTTGATCTGAGAGTTGT
cwp25F	CACTTTGATGAGAGCATGGG
cwp25R	TTAGTTCATATCTTAAGTTA
cwp35F	TGGGGGAGATAGGAGAATAC
cwp35R	ATCAGTAGTAACTTATTTTTTGGGAAAGAC
myb25F	TGGCTATGTATTTTTTCTTCTTCTACAGCT
myb25R	TAGCAGTACAGAGTAATTATTATTTTAGTA
U65F	TTGAGATGACTCGCCTGATTG
U65R	GAAATTCCGAATGACCTGCTC
